# The burden and outcome of nasopharyngeal carcinoma in Sweden

**DOI:** 10.2340/1651-226X.2025.43700

**Published:** 2025-07-21

**Authors:** Evelina Gille, Anders Näsman, Madeleine Helmersson, Elin Marsk, Antti Mäkitie, Lalle Hammarstedt-Nordenvall

**Affiliations:** a Division of Ear, Nose and Throat Diseases, Department of Clinical Sciences, Intervention and Technology, Karolinska Institute and Karolinska Hospital, Stockholm, Sweden; b Department of Oncology-Pathology, Karolinska Institute, Bioclinicum, Karolinska University Hospital, Stockholm, Sweden; c Department of Clinical Pathology, Karolinska University Hospital, Stockholm, Sweden; d Regional Cancer Center West, Western Sweden Health Care Region, Gothenburg, Sweden; e Department of Otorhinolaryngology – Head and Neck Surgery, University of Helsinki and Helsinki University Hospital, Helsinki, Finland; f Research Program in Systems Oncology, Faculty of Medicine, University of Helsinki, Helsinki, Finland

**Keywords:** Nasopharyngeal carcinoma, Epstein-Barr virus, survival, Swedish Head and Neck Cancer Register

## Abstract

**Introduction:**

The purpose of this study is to present the nationwide disease burden and survival of nasopharyngeal carcinoma (NPC) in Sweden. The subcohort from the Stockholm-Gotland region was included to investigate the prevalence of Epstein-Barr virus (EBV) in NPC and to describe pattern of relapse.

**Methods:**

This population-based nationwide study included patients diagnosed with NPC in Sweden during 2008–2021. The series was retrieved from the Swedish Head and Neck Cancer Register. Age at diagnosis, sex, tumor histopathology, stage, treatment intent, treatment, radiation dose, follow-up time, time to relapse, and site of relapse were recorded. The Stockholm-Gotland region series was used to obtain an updated histopathological analysis including EBV status and to analyze site of relapse.

**Results:**

The nationwide study cohort comprised 399 patients, 33% were female. Mean age at diagnosis did not differ between the sexes: 56.3 years for females, 57.5 years for males. Seventy-one percent presented with Stage III or IV. The 5-year overall survival (OS) was 73.2%. In the regional cohort, 73.9% were EBV positive. In the competing risk analysis, the cumulative incidence of distant metastatic relapse was higher than that of local and/or regional relapse at 5 years (18.7% vs. 12.4%). However, the confidence intervals were wide, and the difference should be interpreted with caution.

**Interpretation:**

The survival outcome in our study seems comparable to previous studies in nonendemic countries. There was a high percentage of EBV-positive tumors compared with the previous studies in nonendemic countries.

## Introduction

Nasopharyngeal carcinoma (NPC) is uncommon in the Western world, with an annual incidence of 1/100,000 person-years [1]. According to GLOBOCAN 2022, NPC accounts for 0.6% of all new cancer cases and 0.75% of cancer deaths worldwide each year. Over 120,000 new cases of NPC were reported globally, whereof 83% in Asia [2].

NPC arises from the mucosal epithelium of the nasopharynx. The World Health Organization (WHO) distinguishes three major histological forms, according to the 4th classification: I: keratinizing squamous cell carcinoma (SCC), II: nonkeratinizing SCC with the differentiated and undifferentiated subtypes, and III: basaloid SCC [3]. Keratinizing SCC is more prevalent in low-incidence populations and is associated with poorer outcomes [4]. In contrast, undifferentiated nonkeratinizing SCC accounts for over 95% of NPC cases in high-incidence regions, is strongly linked to Epstein-Barr virus (EBV) infection, and is associated with better survival rates [5]. Keratinizing SCC is more likely than nonkeratinizing SCC to be locally advanced at its presentation and less likely to present with metastasis to regional lymph nodes [6]. Smoking has been identified as a significant risk factor, particularly for the keratinizing subtype [7]. Most of the knowledge of this disease comes from studies conducted in high-incidence regions, where the vast majority of cases are of the EBV-positive subtype [6, 8, 9].

Although NPC is uncommon in Sweden, it has important consequences due to the high morbidity after treatment [10]. In addition, patients with NPC tend to be younger compared with those with other head and neck cancers [11–13], and as a consequence, many NPC patients will be affected by long-term sequelae. Consequently, better knowledge of the disease behavior and long-term outcome in low-incidence areas can lead to more effective and personalized treatment.

The burden of NPC, histopathological subtypes, and viral status in Europe differs between countries and areas [14– 19]. No previous national Swedish studies describing EBV prevalence in NPC exist, and this forms a knowledge gap in the patients’ characteristics, which makes it difficult to compare reports between nonendemic countries.

The main purpose of this study is to investigate the disease burden of NPC in a nonendemic area, secondary aim to report on viral distribution, and assess the site and pattern of failure.

## Materials and methods

This is a population-based nationwide study including all patients diagnosed with NPC in Sweden during 2008–2021. The national cohort was retrieved from the Swedish Head and Neck Cancer Register (SweHNCR). SweHNCR was established in 2008 and includes 98% of the head and neck cancer cases diagnosed annually in Sweden. SweHNCR includes data from the following parameters: Age at diagnosis, treatment (surgery yes/no, radiotherapy yes/no, chemotherapy yes/no), date of first visit to an ENT specialist, date of multidisciplinary tumor board (MDT), date of treatment start, treatment intention (palliative or curative), histology, TNM classification and tumor stage (UICC 7th ed. and since January 1, 2018: UICC 8th ed.), performance status (PS), follow-up data for 5 years, date and site of relapse, date of death, and tumor status at death [20].

Patients with diagnostic ICD codes C11.0–9 for cancer of the nasopharynx and histology codes for SCC or undifferentiated carcinoma were included. Other histological types were excluded. As the national cohort does not include data on histopathological classification or EBV status, comparison was made with the regional cohort, for which histopathological specimens were available for re-analysis. All patients living in the greater Stockholm-Gotland region and receiving treatment at the Karolinska University Hospital (Stockholm, Sweden) were included in the regional cohort.

In order to assess the probability of relapse, a competing risk analysis was performed for all NPC patients diagnosed during 2008–2021 in the regional cohort. The following parameters were recorded: sex, age at diagnosis, histopathological assessment of the tumor, stage of the disease, PS and intention of treatment (palliative or curative), treatment, follow-up time, recurrence, time to recurrence, site of recurrence, and status of last follow-up. Their histopathology specimens were reclassified by an experienced head and neck pathologist (A.N.), and characterization according to the latest WHO classification (5th Ed.) was performed [3].

In addition, all EBV negative samples were assessed for overexpression of p16 by immunohistochemistry (IHC).

### Statistical analyses

To estimate overall survival (OS) the Kaplan‑Meier method was used, and the log-rank test was implemented to detect difference in survival rates. For relative survival (RS), the Pohar-Perme estimator was used to estimate net survival in a RS setting. Net survival using Pohar-Perme estimates cancer-specific survival as if cancer were the only cause of death. Pohar-Perme provides an estimate of cancer mortality that is independent of mortality patterns in the national general population [21]. Death rates of the Swedish population according to age, sex, and calendar year were used for the estimation of the RS. Length of follow-up was estimated with the Kaplan‑Meier estimate of potential follow-up (KM-PF).

For the patients in the regional cohort, the data were cross-referenced with patient charts to validate the information. For this group, a competing risk analysis was performed with three competing events, distant metastatic relapse, local and/or regional relapse, and death of other causes. The cumulative incidence function was employed to obtain subdistribution estimates for each event, and 95% pointwise confidence intervals were computed [22]. In addition, a competing risk analysis was conducted to estimate the probability of relapse (at any site) when accounting for the competing event of death of other causes for EBV-positive tumors compared with EBV-negative ones. Gray’s test was used to test equality of cumulative incidence functions for relapse.

Recurrence was defined as the reappearance of disease occurring more than 6 months after completion of treatment, whereas residual disease was defined as persistent disease identified within 6 months post-treatment.

R statistical software version 4.3.3 was used for the statistical analyses.

The study design was approved by the Swedish Ethical Review Authority (2019-05-27, Dnr: 2019-01933).

## Results

A total of 65 out of the 464 patients were excluded from the national cohort: 11 due to incorrect dates of treatment and 54 due to incorrect histopathological diagnosis.

### Patient and tumor characteristics

The national cohort comprised 399 patients, 67% were male and 33% female. Mean age was 57.1 years (± 15.7SD). The youngest patient was 12 years and the oldest 92 years.

Regarding PS, the majority (*n* = 305, 82.4%) had WHO PS 0, while 29 (7.3%) patients did not have PS recorded. Clinical stage was registered for 379 patients, stage I: 8.4%, stage II: 20.3%, stage III: 36.9%, and stage IV: 34.3% (Table 1).

**Table 1:** Characteristics of the study cohort with nasopharyngeal carcinoma patients in Sweden diagnosed 2008–2021.

Intent of treatment	Overall	Curative*	Palliative	Missing
***N***	399	353	39	
**Sex (%)**				
Men	267 (66.9)	236 (66.9)	27 (69.2)	0.0
Women	132 (33.1)	117 (33.1)	12 (30.8)	
**Age (mean (SD))**	57.1 (15.7)	55.7 (15.5)	67.8 (13.2)	0.0
**Age (median [IQR])**	59.0 [47.5, 67.5]	58.0 [46.0, 66.0]	70.0 [58.5, 79.0]	0.0
**Age group (years) (%)**				
< 20	10 (2.5)	10 (2.8)	0 (0.0)	0.0
20–50	119 (29.8)	115 (32.6)	4 (10.3)	
51–60	86 (21.6)	74 (21.0)	10 (25.6)	
61–70	108 (27.1)	99 (28.0)	6 (15.4)	
71–80	57 (14.3)	44 (12.5)	11 (28.2)	
> 80	19 (4.8)	11 (3.1)	8 (20.5)	
**Performance status (%)**				
WHO 0	305 (82.4)	290 (87.1)	13 (39.4)	7.3
WHO 1	40 (10.8)	34 (10.2)	5 (15.2)	
WHO 2	14 (3.8)	5 (1.5)	9 (27.3)	
WHO 3	7 (1.9)	3 (0.9)	3 (9.1)	
WHO 4	4 (1.1)	1 (0.3)	3 (9.1)	
**T stage (%)**				
T0	3 (0.8)	3 (0.9)	0 (0.0)	2.8
T1	136 (35.1)	130 (37.7)	6 (16.2)	
T2	81 (20.9)	79 (22.9)	2 (5.4)	
T3	72 (18.6)	60 (17.4)	9 (24.3)	
T4	96 (24.7)	73 (21.2)	20 (54.1)	
**N stage (%)**				
N0	86 (21.9)	6 (21.8)	8 (21.6)	1.8
N1	117 (29.8)	110 (31.5)	7 (18.9)	
N2	150 (38.3)	134 (38.4)	14 (37.8)	
N3	39 (9.9)	29 (8.3)	8 (21.6)	
**M stage (%)**				
M0	372 (94.9)	343 (98.3)	23 (62.2)	1.8
M1	20 (5.1)	6 (1.7)	4 (37.8)	
**Stage (%)**				
I	32 (8.4)	31 (9.2)	1 (2.8)	5.0
II	77 (20.3)	76 (22.5)	1 (2.8)	
III	140 (36.9)	134 (39.6)	4 (11.1)	
IV	130 (34.3)	97 (28.7)	30 (83.3)	
**Treatment (%)**				
Chemoradiotherapy	281 (72.2)	271 (77.0)	9 (25.0)	
Radiotherapy only	87 (22.4)	76 (21.6)	11 (30.6)	
Chemotherapy only	7 (1.8)	4 (1.1)	3 (8.3)	2.5
Other treatment	2 (0.5)	1 (0.3)	1 (2.8)	
No treatment	12 (3.1)	0 (0.0)	12 (33.3)	

* One of the patients registered with curative intent of treatment did not have treatment registered but was tumor free after treatment.

### Radio- and chemoradiotherapy

Of the 399 patients, treatment intention was available for 392 (98.2%), and 353 (90.1%) received curatively and 39 (9.9%) received palliatively intended treatment.

In the group with curative treatment intent, 271 (77.0%) patients received both external radiotherapy and chemotherapy. In the same group, brachytherapy was administered for 30 (8.5%) patients, and the median dose was 8 Gy, mean 8.0 (SD ± 1.0). In total, 347 patients in this group received external radiotherapy, and the median radiation dose was 68 Gy, and the mean was 67.3 Gy (SD ± 6.7). Altogether, 275 (78.1%) patients in the group received curative intent chemotherapy*.*

Thirty-nine (9.9%) patients received palliative treatment. Eleven of them received only radiotherapy, three patients only medical treatment, nine patients received radiotherapy and medical treatment, and one patient received surgery. Twelve patients received no treatment. Missing information of the treatment for three patients with palliative intent of treatment.

### Survival

All patients with curative intent of treatment were included in the survival analysis. Median follow-up time was 77.2 months (IQR 41.6–131.3).

**Figure 1:**
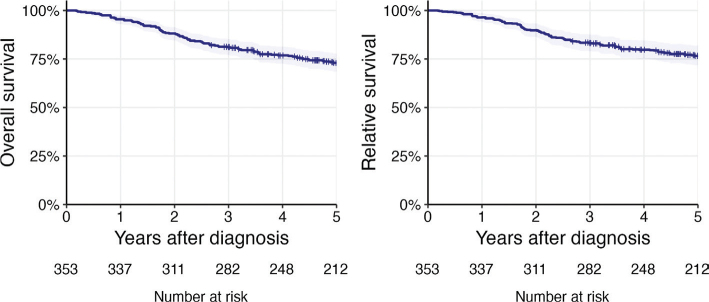
Five-year overall survival and 5-year relative survival for nasopharyngeal carcinoma patients in Sweden diagnosed 2008–2021.

The 5-year OS for patients treated with curative intent was 73%, (95% CI 68.4–77.8). The 5-year RS was 76.6% (95%CI 71.7–81.8) (Figure 1).

For patients with stage I‑II disease, the 5-year OS was 83.6% (95% CI 76.8––91.1), for stage III, 69.3% (95% CI 61.8– 77.8), and for stage IV 68.6% (95% CI 59.8–78.6).

The 5-year OS and the median OS for the patients receiving treatment with palliative intent was 17.6% (95% CI 8.8–35.0) and 15.1 months (95% CI 6.5–25.4), respectively.

### Regional cohort

Among the 88 patients in the regional cohort, 64% were male and 36% female.

In total, 70 (79.5%) histological specimens were reviewed. 18 specimens were not available from the biobank. Overall, 73.9% were EBV positive. The majority of the patients in the regional cohort were diagnosed with the undifferentiated subtype of nonkeratinizing SCC (36 patients, 51.4%), of whom 94.4% were EBV positive. A total of 28 patients (40%) had the differentiated subtype of nonkeratinizing SCC, with 60.7% of this group being EBV positive. Six patients (8.7%) had keratinizing SCC, and none in this group were EBV positive (Table 2).

**Table 2:** Characteristics of the cohort, nasopharyngeal carcinoma patients in the Stockholm-Gotland region diagnosed 2008–2021.

Histology	Overall	Keratinizing squamous cell carcinoma	Nonkeratinizing squamous cell carcinoma -differentiated	Nonkeratinizing squamous cell carcinoma-undifferentiated	Missing
**n**	88	6	28	36	
**Sex (%)**					
Men	56 (63.6)	5 (83.3)	17 (60.7)	24 (66.7)	0.0
Women	32 (36.4)	1 (16.7)	11 (39.3)	12 (33.3)	
**Age (mean (SD))**	53.9 (15.2)	61.5 (14.5)	59.0 (9.7)	48.6 (17.5)	0.0
**Age (median [IQR])**	56.0 [46.8, 63.2]	58.5 [51.5, 70.8]	58.5 [53.8, 64.0]	51.5 [41.0, 59.8]	0.0
**Age group (years) (%)**					
< 20	5 (5.7)	0 (0.0)	0 (0.0)	5 (13.9)	0.0
20–50	29 (33.0)	2 (33.3)	6 (21.4)	12 (33.3)	
51–60	26 (29.5)	1 (16.7)	11 (39.3)	10 (27.8)	
61–70	18 (20.5)	1 (16.7)	8 (28.6)	7 (19.4)	
71–80	8 (9.1)	1 (16.7)	2 (7.1)	2 (5.6)	
> 80	2 (2.3)	1 (16.7)	1 (3.6)	0 (0.0)	
**Performance status (%)**					
WHO 0	72 (84.7)	4 (66.7)	24 (88.9)	31 (86.1)	3.4
WHO 1	9 (10.6)	2 (33.3)	3 (11.1)	3 (8.3)	
WHO 2	3 (3.5)	0 (0.0)	0 (0.0)	(2.8)	
WHO 3	1 (1.2)	0 (0.0)	0 (0.0)	1 (2.8)	
**Intent of treatment (%)**					
Curative	81 (92.0)	6 (100.0)	25 (89.3)	34 (94.4)	0.0
Palliative	7 (8.0)	0 (0.0)	3 (10.7)	2 (5.6)	
**T stage (%)**					
T0	0 (0.0)	0 (0.0)	0 (0.0)	0 (0.0)	2.3
T1	26 (30.2)	0 (0.0)	8 (29.6)	9 (25.0)	
T2	16 (18.6)	2 (33.3)	4 (14.8)	9 (25.0)	
T3	24 (27.9)	1 (16.7)	9 (33.3)	12 (33.3)	
T4	20 (23.3)	3 (50.0)	6 (22.2)	6 (16.7)	
**N stage (%)**					
N0	17 (19.3)	3 (50.0)	6 (21.4)	4 (11.1)	0.0
N1	33 (37.5)	1 (16.7)	10 (35.7)	15 (41.7)	
N2	35 (39.8)	2 (33.3)	10 (35.7)	16 (44.4)	
N3	3 (3.4)	0 (0.0)	2 (7.1)	1 (2.8)	
**M stage (%)**					
M0	82 (93.2)	6 (100.0)	24 (85.7)	34 (94.4)	0.0
M1	6 (6.8)	0 (0.0)	4 (14.3)	2 (5.6)	
**Stage (%)**					
I	4 (4.7)	0 (0.0)	0 (0.0)	2 (5.6)	2.3
II	18 (20.9)	0 (0.0)	6 (22.2)	9 (25.0)	
III	37 (43.0)	3 (50.0)	11 (40.7)	16 (44.4)	
IV	27 (31.4)	3 (50.0)	10 (37.0)	9 (25.0)	
**Treatment (%)**					
Chemoradiotherapy	71 (83.5)	4 (80.0)	22 (81.5)	32 (88.9)	
Radiotherapy only	9 (10.6)	1 (20.0)	3 (11.1)	3 (8.3)	
Chemotherapy only	2 (2.4)	0 (0.0)	1 (3.7)	1 (2.8)	3.4
Other treatment	0 (0.0)	0 (0.0)	0 (0.0)	0 (0.0)	
No treatment	3 (3.5)	0 (0.0)	1 (3.7)	0 (0.0)	
**EBV (%)**					
EBV-	18 (26.1)	5 (100.0)	11 (39.3)	2 (5.6)	21.6
EBV+	51 (73.9)	0 (0.0)	17 (60.7)	34 (94.4)	
**P16 (%)**					
Negative	39 (72.2)	3 (50.0)	11 (55.0)	25 (89.3)	38.6
Positive	15 (27.8)	3 (50.0)	9 (45.0)	3 (10.7)	
**HPV (%)**					
Negative	9 (47.4)	2 (50.0)	4 (40.0)	3 (60.0)	78.4
Positive	10 (52.6)	2 (50.0)	6 (60.0)	2 (40.0)	

EBV: Epstein-Barr virus.

Five patients in the regional cohort were under the age of 20 years, and they all had an undifferentiated nonkeratinizing SCC that was EBV positive.

Clinical stage was registered for 86 patients, 4.7% of whom presented with stage I, 20.9% with stage II, 43% with stage III, and 31.4% with stage IV.

In the regional cohort, 80 (92%) patients were treated with curative intent, and seven (8%) with palliative intent. The relative 5-year survival for the patients treated with curative intent in the regional cohort was 73.6% (95% CI 63.9–84.8), compared with 77.4% (95%CI 71.8–83.5) in the national cohort with the regional cohort excluded (log-rank test, *p*-value 0.537).

**Figure 2:**
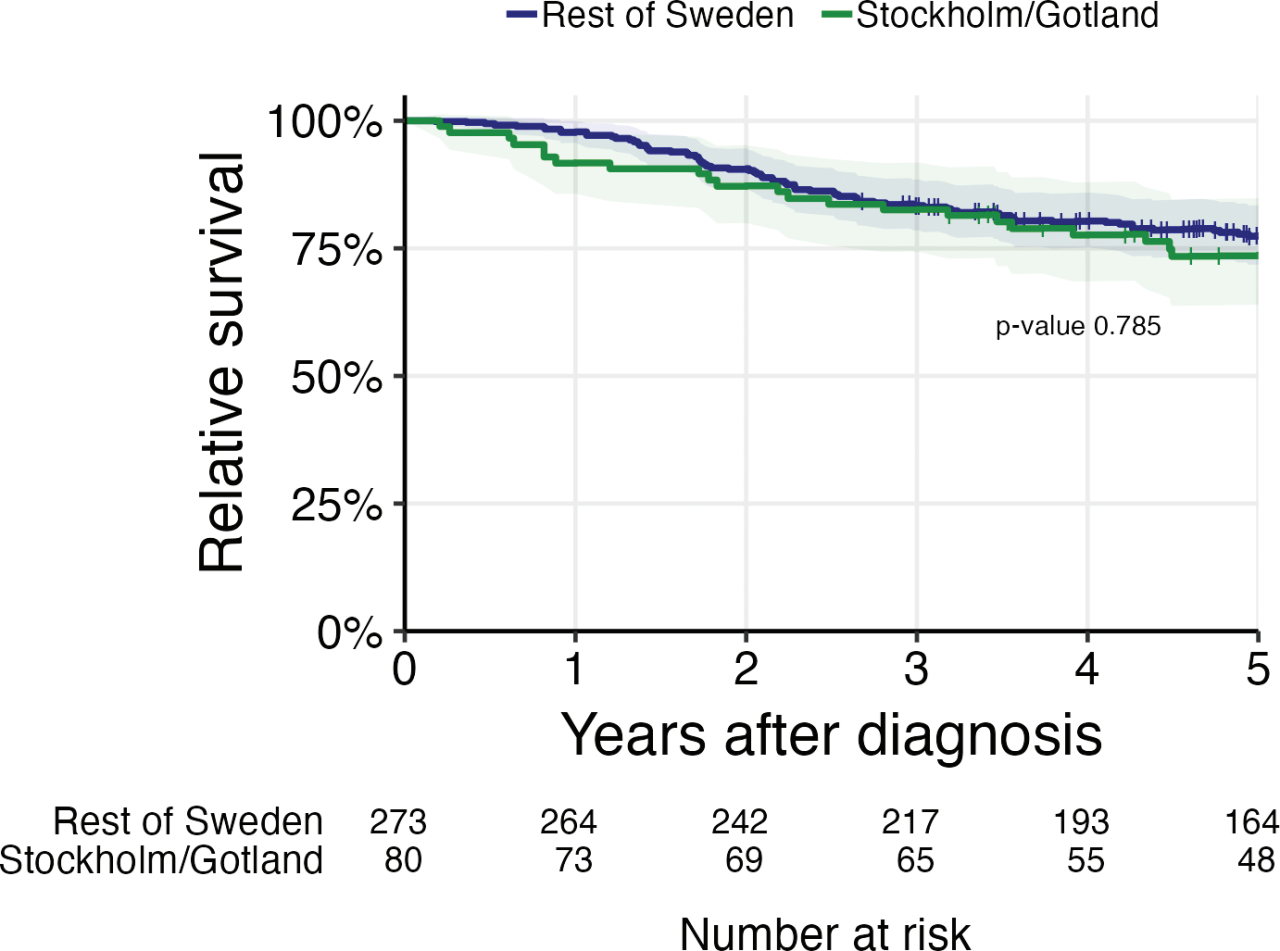
Relative survival for nasopharyngeal carcinoma (NPC) patients in Sweden compared with NPC patients in the Stockholm-Gotland subcohort diagnosed 2008–2021.

Five-year OS for patients in the regional cohort was 50% (95% CI 22.5–100.0) for keratinizing SCC, 65.9% (95% CI 49.0–88.5) for differentiated nonkeratinizing SCC, and 72.8% (95%CI 59.0–89.7) for undifferentiated nonkeratinizing SCC. Altogether, the 5-year OS for patients in this cohort was 72.5% (95% CI 63.1–83.3) (Figure 2).

Relative 5-year survival for patients in the regional cohort was 52.8% (95% CI 26.1–106.9) for KSCC, 67.7% (95% CI 50.4–90.8) for differentiated nonkeratinizing SCC, and 76.7% (95% CI 63.0–93.3) for undifferentiated nonkeratinizing SCC.

**Figure 3:**
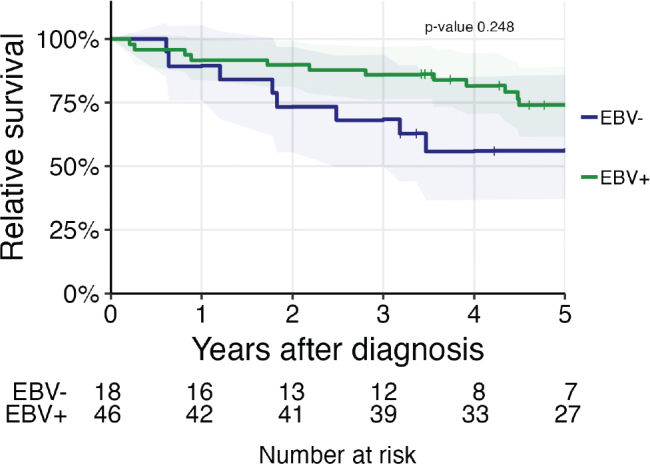
Relative survival for nasopharyngeal carcinoma patients in the Stockholm-Gotland region diagnosed 2008–2021. EBV-positive cases were compared with the EBV-negative ones. EBV: Epstein-Barr virus.

Five-year OS for patients given treatment with curative intent was 54.3% (95% CI 35.2–83.9) for those with an EBV-negative tumor and 72.5% (95% CI 60.3–87.2) for those with an EBV-positive tumor. But the difference in 5-year OS between EBV-positive and EBV-negative tumors is not significant (*p*-value 0.094) (Figure 3).

Human papillomavirus (HPV) status was assessed in 13 out of the 18 (5 specimens contained insufficient remaining material for HPV DNA analysis) patients who were negative for EBV testing by in situ hybridization.

Ten were HPV positive, and three were HPV negative. Among the 10 HPV-positive tumors nine were also positive for p16, defined as > 70% positive p16 staining cells.

Altogether, 76 patients diagnosed in the regional cohort and treated with curative intent of treatment were included in the competing risk analysis. Four patients were excluded due to not being tumor free after treatment or for missing tumor status.

**Figure 4:**
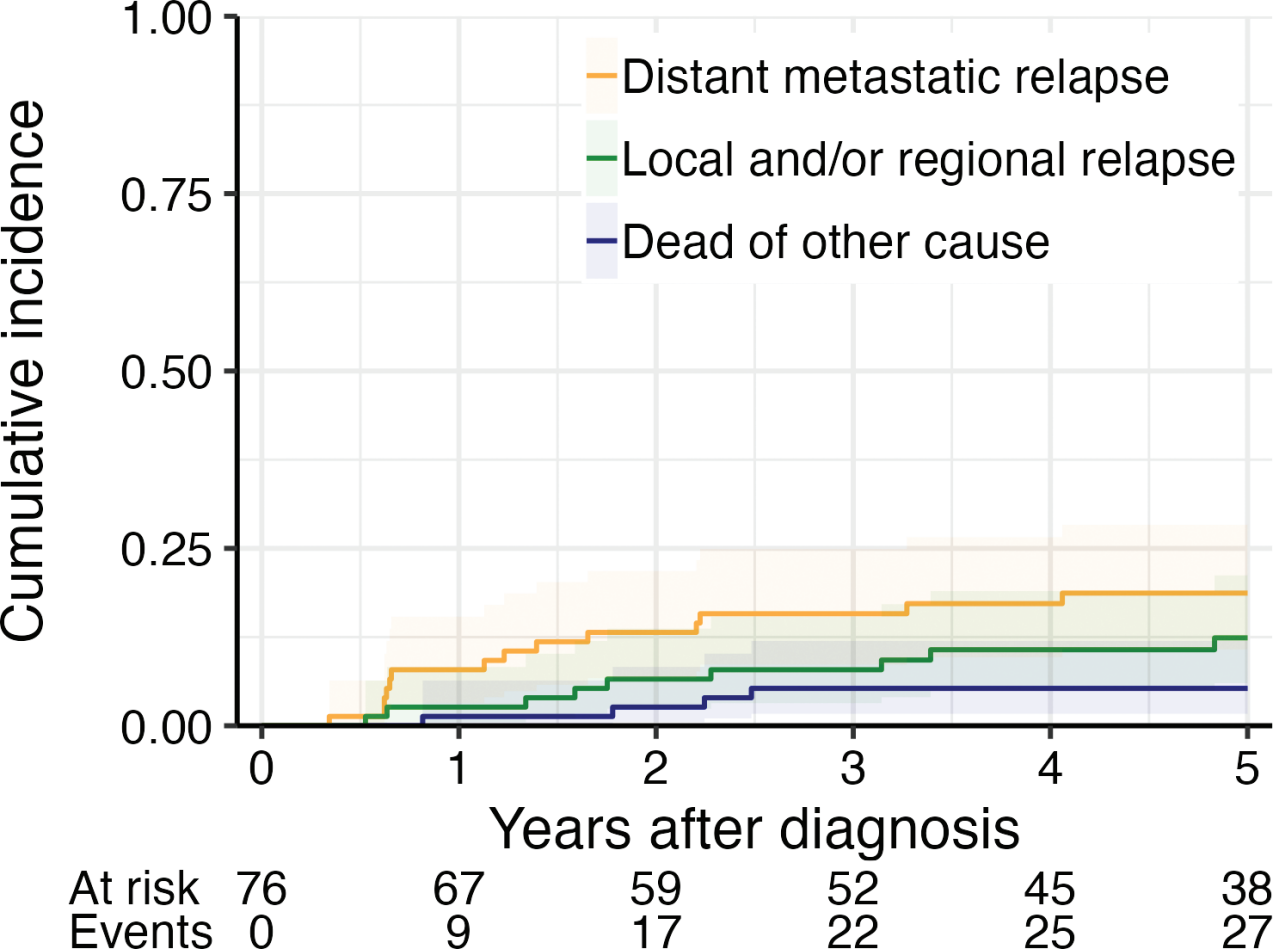
Competing risk: Probability of event in percent (cumulative incidence) for dying without relapse and having relapse for nasopharyngeal carcinoma patients in the Stockholm-Gotland region diagnosed 2008–2021.

In the competing risk analysis for distant relapse or local and/or regional relapse, the cumulative incidence of having a distant relapse as the first relapse was 18.7% (95% CI 10.8–28.3%) at 5 years after diagnosis. The cumulative incidence of local and/or regional relapse at 5 years was 12.4% (95% CI 6.0–21.2) (Figure 4).

In the competing risk analysis stratified by EBV status, there was no difference between the two groups in risk for relapse or in death without relapse (*P*-value > 0.9, Gray’s test).

## Discussion

This study presents epidemiological data on NPC in a nonendemic area. We compared the survival of patients diagnosed with NPC during 2008–2021 in the regional cohort with those in the national Swedish population-based register. The regional cohort, which includes reviewed individual histopathological samples and information on EBV status, was included to further investigate the prevalence of EBV association of the Swedish NPC. No significant difference in survival was observed between the regional and national cohort with the regional cohort excluded, suggesting that the regional cohort is representative of the nationwide NPC patient population*.* The key findings of this study include an OS comparable to reports from other nonendemic countries. As a secondary finding, the relatively high proportion of EBV-positive tumors in this nonendemic country aligns with results from previous studies [14, 16–19, 23].

Further, a small proportion of the patients had an HPV-positive tumor. Additionally, in most patients a distant site of relapse was observed, in line with previously published data on EBV-positive tumors [24–27]. The proportion of distant relapse is high compared with other head and neck cancers [28]. However, similar prevalences of EBV positivity were reported also from other regions in Sweden [16].

There are divergent results according site of relapse. Several studies from endemic regions such as Hong Kong, Guangdong Province in China, Saudi Arabia have reported distant metastasis as the most common site of relapse [24–27]. This is in contrast to a study from another nonendemic country, Finland, where locoregional relapse was the most common site of relapse [15].

There has been a small number of reports published on the distribution and outcome of NPC in the Western world. A study from the United Kingdom showed a higher percentage of nonkeratinizing tumors and also distant metastasis as the most common site of recurrence [29], which is in line with studies from endemic regions. Likewise, an Italian international multicenter analysis of NPC in nonendemic regions presented survival commensurate with endemic countries [30], and a large study from the Netherlands showed decreased incidence of NPC but increased incidence of nonkeratinizing tumors in the same time period [31]. However, these data may not directly be transferable to the Swedish population. Speculatively, the population in Sweden might differ from the other European countries in the number of immigrants. There are at present limited data on the disease and its outcome in a nationwide setting, and the general knowledge of the disease in this part of the world is sparse, and these publications do not specifically address the situation in Sweden.

In the present cohort, the 5-year OS was 73%, which is, as expected, lower than 78.2% in a Hong-Kong series [24] but higher than 56% in the Danish study, including patients from 2000–2018 [14]. The 5-year OS in the aforementioned Finnish series [15] was lower (56%) than in the present study, but given the fact that the study comprised a series from 1990 to 2010 and reported increasing survival rate during that study period, there is a reason to believe that this difference is unlikely to be of clinical relevance.

Regarding excess mortality as described by RS, our data show a higher survival than the study from the Netherlands [31]. Our study revealed a 5-year OS of 73.2%, and 5-year RS of 76.8% regardless of stage, whereas the Dutch study revealed a RS of 55%, regardless of stage. This might be due to the fact that different time periods were analyzed or the fact that the background populations are not fully comparable. Several studies indicate increasing 5-year OS and decreasing NPC incidence as a result of improvements in diagnostics and treatment, reduced smoking, dietary changes, and economic development [5, 31, 32].

Our study showed a 73.9% EBV positivity in the tumors in the regional cohort, which confirms results in earlier similar studies [13, 15–18, 20]. There was no difference in survival between the regional cohort and the national cohort, and this may thus indicate a similar distribution of EBV positivity in the rest of Sweden.

The viral distribution in European countries is divergent. There are previous studies that describe virus prevalence in Finland, Denmark [14, 15], and also in southern Sweden. The Finnish study showed 62% of the tumors to be EBV positive, and the Danish study reported 72% EBV positivity in a series of 221 patients. The study from southern Sweden included 48 patients, whereof 75% were EBV positive [15, 16]. In a study from the Czech Republic and Slovakia, EBV was detected in 85.5%. Data from a Turkish study showed 87% EBV positivity [18], and those from the United Kingdom showed 67% EBV positivity [19].

HPV is well established as an agent for developing oropharyngeal carcinoma, and there is an ongoing discussion about the role of HPV in NPC in nonendemic areas. A recent review article showed a global prevalence of HPV positivity in NPC of 18% (95% CI 14–23), with the highest prevalence in North America (25%, 95% CI 17–36) and the lowest prevalence in Asia (13%, 95%CI 8–22) [33].

Our study has several limitations. Firstly, information on ethnicity is not recorded in the Swedish hospital records, which restricts our ability to assess potential ethnic differences in disease presentation or outcomes. Furthermore, the limited size of the regional cohort reduces the statistical power of the analyses. Additionally, a new histopathological classification, introduced in 2017 [34], was not uniformly applied throughout the study period (2008–2021), which could introduce inconsistencies in the classification of cases in the nationwide SweHNCR cohort. The SweHNCR has only been registering chemotherapy use as a dichotomous variable (yes/no) until 2023 when the parameters regarding timing and setting (induction/concurrent/adjuvant) as well as immunotherapy treatment were introduced. Moreover, the data in the SweHNCR are not reported according to the WHO classification, which may limit comparability with other international studies. Lastly, our study may not fully capture the variability in treatment strategies or their impact on survival outcomes, highlighting the need for further research involving larger cohorts and more comprehensive evaluations of treatment protocols.

This population-based study provides valuable insights into the burden of NPC in Sweden, a nonendemic area, and offers an extensive analysis of patient outcomes. NPC, although rare in the Western world, is of significant clinical importance due to its high morbidity and the long-term sequela associated with treatment, especially when considering the relatively young population at risk. We conclude that this population-based study from a nonendemic country demonstrates a relatively high percentage of EBV-positive tumors, which confirms earlier similar studies. Interestingly, our data revealed distant metastasis to be the most common failure site.

## Supplementary Material



## Data Availability

The data underlying this study were provided by the Swedish Head and Neck Cancer Registry (SweHNCR) and the Regional Cancer Center Väst. Data are available from the corresponding author upon reasonable request with the permission of SweHNCR.
