# Supplementary material for: The burden and outcome of nasopharyngeal carcinoma in Sweden
Source: Acta Oncol. 2025 Jul 21;64:43700. doi: 10.2340/1651-226X.2025.43700 (PMC12305471; doi:10.2340/1651-226X.2025.43700)
Supplement: Supplementary file 1 [file AO-64-43700-s1.pdf]

Supplementary material has been published as submitted. It has not been copyedited, or typeset by Acta Oncologica

Method, histopathology

Description of the analysis technique:

Briefly, the ready-to-use antibody p16 (CINtec®, Roche) was used after a Heat Induced Epitope Retrieval (HIER), using CC1 solution for 48 min. The incubation with primary antibody was 24 min. Presence of EBV was assessed using standard protocol with INFORM EBER Probe (Roche) together with ISH Protease 3 (Roche), Red Counterstain II (Roche) and RNA Positive Control Probe (Roche). All stainings were performed on the Ventana BenchMark Ultra platform. In addition, p16-positive cases were analyzed for presence of HPV DNA with the BD Onclarity™ HPV Assay (BD).
